# Novel Anti-Metastatic Action of Cidofovir Mediated by Inhibition of E6/E7, CXCR4 and Rho/ROCK Signaling in HPV^+^ Tumor Cells

**DOI:** 10.1371/journal.pone.0005018

**Published:** 2009-03-26

**Authors:** Abdessamad Amine, Sofia Rivera, Paule Opolon, Mehdi Dekkal, Denis S. F. Biard, Hakim Bouamar, Fawzia Louache, Michael J. McKay, Jean Bourhis, Eric Deutsch, Marie-Catherine Vozenin-Brotons

**Affiliations:** 1 Laboratoire UPRES EA 27-10 Radiosensibilité des tumeurs et tissus sains, Institut Gustave Roussy/Institut de Radioprotection et de Sureté Nucléaire, Villejuif, France; 2 UMR 8121 Laboratoire de vectorologie et transfert de gènes, Institut Gustave Roussy, Villejuif, France; 3 CEA-DSV-iRCM / INSERM U935. Institut A. Lwoff-CNRS, BP 8, Villejuif, France; 4 INSERM U 790, Institut Gustave Roussy, Villejuif, France; 5 Australian National University and The Canberra Hospital, Canberra, Australia; University of Birmingham, United Kingdom

## Abstract

Cervical cancer is frequently associated with HPV infection. The expression of E6 and E7 HPV oncoproteins is a key factor in its carcinogenicity and might also influence its virulence, including metastatic conversion. The cellular mechanisms involved in metastatic spread remain elusive, but pro-adhesive receptors and their ligands, such as SDF-1α and CXCR4 are implicated. In the present study, we assessed the possible relationship between SDF-1α/CXCR4 signaling, E6/E7 status and the metastatic process. We found that SDF-1α stimulated the invasion of E6/E7-positive cancer cell lines (HeLa and TC-1) in Matrigel though CXCR4 and subsequent Rho/ROCK activation. In pulmonary metastatic foci generated by TC-1 cells IV injection a high proportion of cells expressed membrane-associated CXCR4. In both cases models (*in vitro* and *in vivo*) cell adhesion and invasion was abrogated by CXCR4 immunological blockade supporting a contribution of SDF-1α/CXCR4 to the metastatic process. E6 and E7 silencing using stable knock-down and the approved anti-viral agent, Cidofovir decreased CXCR4 gene expression as well as both, constitutive and SDF-1α-induced cell invasion. In addition, Cidofovir inhibited lung metastasis (both adhesion and invasion) supporting contribution of E6 and E7 oncoproteins to the metastatic process. Finally, potential signals activated downstream SDF-1α/CXCR4 and involved in lung homing of E6/E7-expressing tumor cells were investigated. The contribution of the Rho/ROCK pathway was suggested by the inhibitory effect triggered by Cidofovir and further confirmed using Y-27632 (a small molecule ROCK inhibitor). These data suggest a novel and highly translatable therapeutic approach to cervix cancer, by inhibition of adhesion and invasion of circulating HPV-positive tumor cells, using Cidofovir and/or ROCK inhibition.

## Introduction

Tumor cell metastasis remains the main cause of death in cancer patients. The mechanisms involved in the formation of secondary tumors at distant sites are complex. Several molecular determinants are required to proceed through the “active” steps of the metastatic process, such as escape from anoikis/apoptosis, preferential homing to specific organs, extravasation and growth in such secondary sites. Numerous target molecules have been implicated in organ-specific tumor metastasis but its detailed mechanism remains obscure. Current therapeutic options for metastatic cancer are largely limited to classical cytotoxic agents in addition to endocrine manipulation for hormone-sensitive cancers [1].

Specific extracellular chemokines play critical roles in determining organ-selective metastasis, altering chemo-attraction, adhesion and tumor cell survival. Among them, stromal-derived factor-1 (SDF-1α or CXCL12) and its receptor, CXCR4, have been increasingly studied [2]. SDF-1α is a small secreted peptide belonging to the CXC family that regulates migration of leukocytes, especially during immune and inflammatory reactions [3], [4]. The SDF-1α receptor (CXCR4/CD186) is a G-protein coupled receptor with various physiological functions such as leukocyte trafficking, B-cell lymphopoiesis, myelopoiesis, and gastrointestinal vascularisation [5], [6]. The interaction between SDF-1α and CXCR4 can elicit a direct chemo-attractive action on breast cancer cells [7], [8] and CXCR4 expression currently used in conjunction with CD133 to define metastatic stem cells [9] suggesting that its pro-metastatic action could broadly apply to metastatic cancer including cervical carcinoma. SDF-1α protein is indeed highly synthesized at common metastatic sites, including bone marrow, liver and lung [10] and CXCR4 is highly expressed on cervix cancer cells [11]. It has been suggested that ligand-receptor interaction and subsequent activation of the SDF-1α/CXCR4 complex may preferentially arrest cancer cells in the vascular beds of SDF-1α−expressing organs and could contribute to metastatic homing and invasion of these cells in such specific organs [2]. The functional consequences of SDF-1α/CXCR4 axis activation include modulation of cell motility, adhesion and invasion dependent on actin cytoskeleton dynamics [12], [13]. Such phenotypic alterations are triggered, at least in part, by the small GTPases RhoA, rac1 and cdc42 [14] and activation of their downstream effectors, Rho kinases/ROCK/ROK, leading to myosin phosphorylation [15], [16], [17], [18], [19].

One causative agent in cervix carcinogenesis is infection with high risk human papilloma viral subtypes, including HPV-16 and -18 [20]. Expression of the oncoprotein, E6 and E7, have an established role in the initiation and growth of cervix cancer mediated by the inhibition of the products of the tumor suppressor genes p53 and pRB [21]. Human HPV genotype has some prognostic value in early-stage cervical cancers [22]; conversely positive HPV status correlates with reduced tumour aggressiveness and improved patient outcome, especially in Head & Neck tumors [23]. Although a causal aetiology for oncogenic HPV in metastatic spread remains an open question, several lines of evidence support its possible role in this process. For example, isogenotypic HPV DNA has been found in primary tumours and metastatic lymph nodes in the same patients [24], [25]; stable transfection of HPV-16 E6 and E7 genes increased the pro-metastatic conversion in two non-isogenic mouse cell lines (NCTC2555 and NIH3T3) [26]; and HPV-16 E6/E7 enhanced migration of normal human keratinocytes [27] and promoted cell invasion and metastasis of human breast cancer cells [28].

Blood-born metastasis of cervical cancer are not uncommon especially in third world countries, prompting us to investigate the relationship between the SDF-1α/CXCR4 axis, the HPV-16/18-E6/E7 genes, and tumor metastasis. We show that SDF-1α stimulates HPV-positive cell lines (HeLa and TC-1) invasiveness both *in vitro* and *in vivo* and this was mediated though CXCR4 and subsequent Rho/ROCK activation. We then show that inhibition of E6 and E7 by knock-down approach and a pharmacological anti-viral agent, Cidofovir (HPMPC, VISTID®) [29] strongly abrogated SDF-1α-induced invasion, decreased CXCR4 expression *in vitro* and inhibited formation of lung metastasis. Based on these data, we propose a new pharmacological approach to inhibit metastasis in cervical cancer patients through the inhibition of E6/E7 with Cidofovir and ROCK.

## Results

### CXCR4 immunological blockade inhibits cell invasion *in vitro*

**Figure 1:**
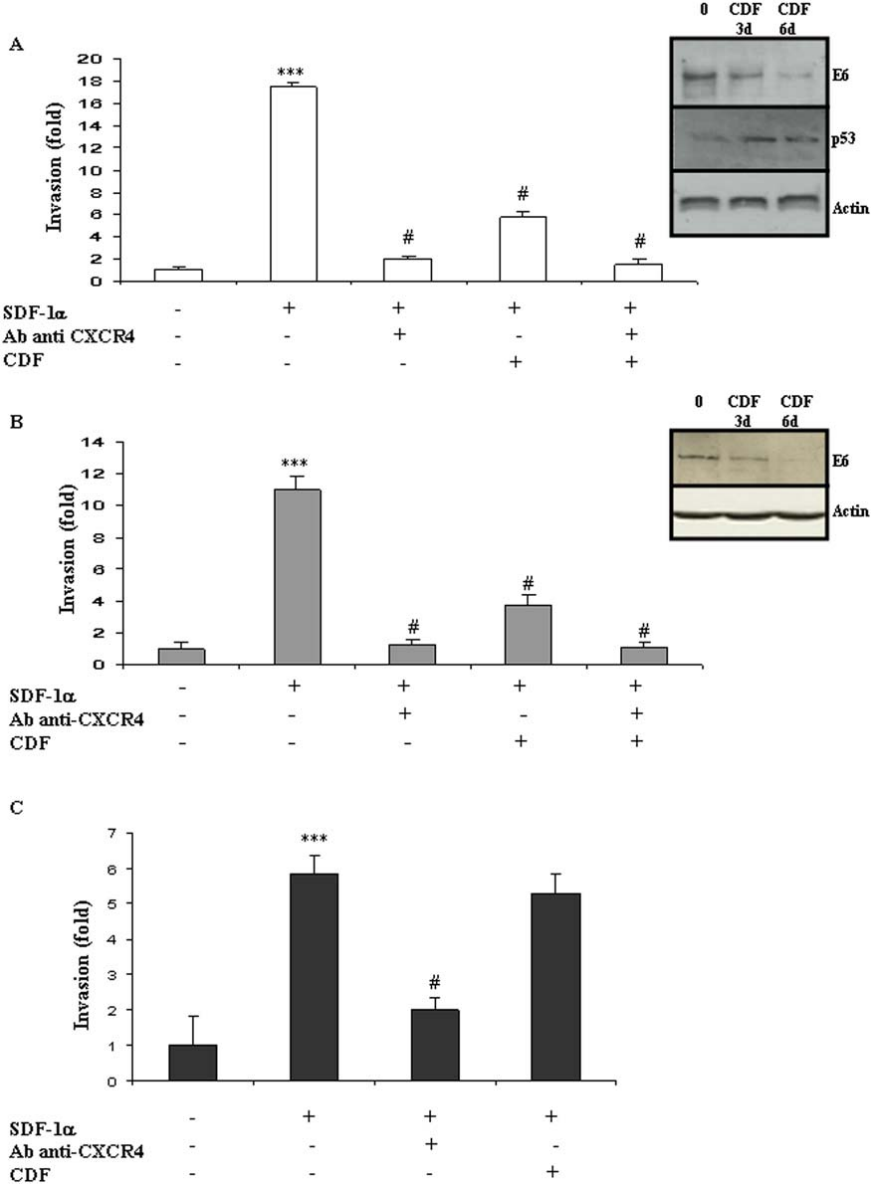
*In vitro* cell invasion is stimulated by the SDF-1/CXCR4 pathway independently from HPV status. The modulation of E6 expression was monitored using Western-blot in HPV-positive HeLa (A) and TC-1 (B) cells and in HPV-negative B16F10 (C) cells, after 3 and 6 days of incubation with Cidofovir (CDF). Modulation of P53 expression was assessed in HeLa cells after CDF incubation (A). Cell invasion was measured using a Matrigel assay in HeLa (A), TC-1 (B) and B16F10 (C) cells. Recombinant human CXCL12/SDF-1 (100 ng/mL; R&D Systems) was used as chemoattractant and modulation of cell migration was recorded after treatment with CXCR4-blocking antibody or/and Cidofovir (CDF). The invasion rate was determined by counting crystal violet-stained cells. Invasion was stimulated by SDF-1α/CXCR4 independently from the HPV status of the cells but Cidofovir anti-invasive action was restricted to the two HPV-positive cell lines. Three independent experiments with three chambers each time were performed. ***P<0.01, for a statistically true difference, as compared to the untreated group. # *P*<0.01 compared to SDF-1α-treated group.

To determine a potential role for the SDF-1/CXCR4 axis in cells' invasiveness, SDF-1α was used as chemoattractant on the two HPV-positive cell lines HeLa and TC-1 and one HPV-negative cell line B16F10. SDF-1α at 100 ng/ml significantly enhanced invasion in the three cell types tested (Figure 1A, B, C) (*** *P*<0.01). CXCR4 was then blocked using a specific monoclonal blocking antibody. This blocking antibody markedly suppressed cell invasion in all three cell types (10 µg/mL; *# P*<0.01).

### E6 and E7 inhibition by Cidofovir treatment and KD blocks cell invasion *in vitro*

**Figure 2:**
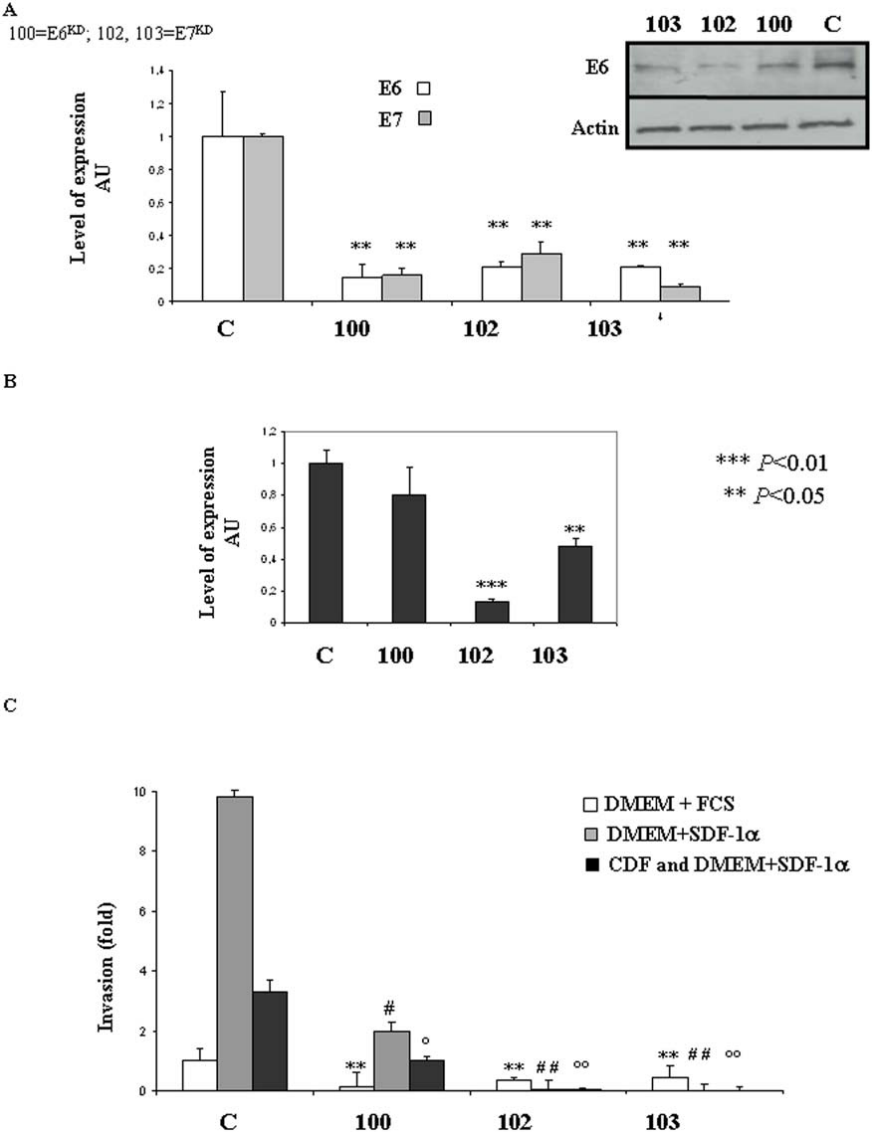
Silencing of E6 and E7 showed contribution of the oncoproteins to the pro-migratory phenotype of HeLa cells. (A) Quantitative RT-PCR analysis of E6 and E7 mRNA level and E6 protein expression in knock-down HeLa cells lines. E6 and E7 expression were respectively knocked-down using the pEBVsiRNA in 100, and 102, 103. Inhibition was statically significant *P*<0.04. (B) Quantitative RT-PCR analysis of CXCR4 mRNA level in clones^KD^ with CXCR4- specific probe. ***P<0.01 and **P<0.05. (C) Cell invasion was measured using a Matrigel assay in E6^KD^ (100) and E7^KD^ (102, 103) and compared to migration of a control HeLa clone (scrambled sequence). All Experiments were conducted in presence of Hygromycin (125 µg/mL) with DMEM+10% FCS; DMEM+SDF-1α (100 ng/mL); CDF and DMEM+SDF-1α (100 ng/mL). Three independent experiments with three chambers each time were performed. **P<0.05, for a statistically true difference, as compared to the control clone with DMEM+FCS. # *P*<0.05 and ## *P*<0.01 compared to the control clone with DMEM+SDF-1α. ° *P*<0.05 and °° *P*<0.01 compared to the control clone with CDF and DMEM+SDF-1α.

We next examined whether the presence of HPV oncoproteins could contribute to the invasiveness of HeLa and TC-1 cells using the E6/E7 inhibitor, Cidofovir (CDF). Western-blot confirmed E6 down-regulation in HeLa and TC-1 cells treated by Cidofovir (Figure 1A, B WB panels). Six days of incubation with Cidofovir inhibited E6 more potently than 3 days and these conditions were therefore used in subsequent experiments (Figure 1A, B). In HeLa cells E6 inhibition was associated with P53 restoration (Figure 1A). To exclude potential non-specific drug cytotoxicity, we next determined the effect of Cidofovir on cell survival and apoptosis. Cidofovir treatment reduced HeLa cell survival by 10% (ns) and TC-1 survival by 20% (ns), as measured by dimethylthiazol diphenyl tetrazolinuim (MTT) assay (Figure S1A). Consistently, Cidofovir treatment increased apoptosis in HeLa cells by 8% and in TC-1 by 20% (p<0.05) (Figure S1B). These measurements were used to adjust the number of seeded cells, to ensure a reliable comparison between groups. In each group, an expected 5×10^5^ living cells were seeded into the top chamber of a Matrigel transwell. HPV-negative cells B16F10 were used as controls. Cidofovir significantly abrogated the SDF-1α-induced invasiveness of HPV-postive cells *i.e.* HeLa and TC-1 (Figure 1A, B; #*P*<0.01) but had no inhibitory effect on HPV-negative cells *i.e.* B16F10 (Figure 1C), thereby suggesting that the capacity of Cidofovir to modulate invasiveness is dependant on HPV status. To further investigate possible role of E6 and E7 oncoproteins in the metastatic process the two oncoproteins were knocked-down (KD) in HeLa cells using a method recently described by Biard [30]. This stable KD approach was used because of the multiple copy of E6/E7 insertion in the genome of HeLa cells making traditional knock-out experiments non-viable. Three independent clones (100, 102, 103) were produced and showed approximately 80% inhibition of the targeted genes as shown in Q-RT-PCR and Western-blot (Figure 2A). This inhibition rate is stable in time (15 passages tested so far). Targeting E6 resulted in E6 and E7 switch-off and *vice et versa* as already described [31], [32] and due to their common ORF. CXCR4 gene expression was reduced by 90% in the clone 102 (p<0.01), by 50% in the clone 103 (p<0.05) while a trend toward reduction was obtained in the clone 100 (Figure 2B). Cell invasion was then monitored using matrigel assays in presence of SDF-1α or not and with clones pre-treated or not with Cidofovir (CDF) (Figure 2C). In the three E6/E7 KD clones (100, 102, 103) constitutive migratory activity was reduced (p<0.05). In addition, SDF-1α pro-migratoy action was impaired in the three KD clones. In the clones 102 and 103, SDF-1α was unable to stimulate cell migration (Figure 2C) consistently the reduced CXCR4 gene expression measured by Q-RT-PCR (Figure 2B). In the clone 100, SDF-1α stimulated cell migration consistently with the persistent CXCR4 gene expression found in this clone (Figure 2B). Cidofovir treatment was then performed on the three KD clones. In the clone 102 and 103, Cidofovir had no additional effect as cell migration was already abrogated in these clones. However, Cidofovir reduced cell migration in the clone 100 as compared to control clone. Collectively, these results support a direct involvement of E6 and E7 proteins in the invasiveness of HeLa cells in our experimental conditions.

### CXCR4 inhibition blocks experimental metastasis *in vivo*

**Figure 3:**
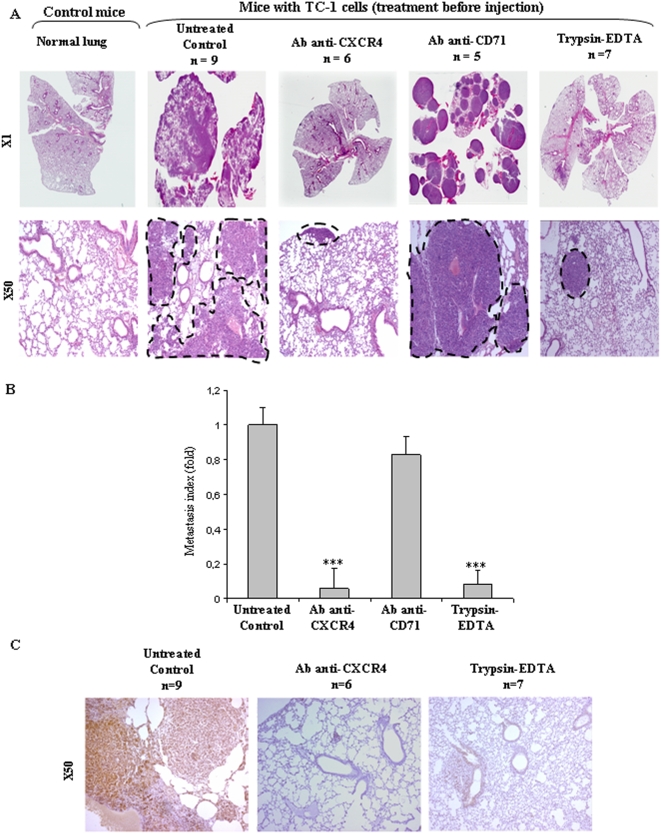
TC-1 cell lung metastasis is mediated by the CXCR4 *in vivo.* TC-1 cells (1×10^6^) were injected into mice tail veins either without or with pre-treatment with CXCR4-blocking antibody (2B11), irrelevant antibody (CD71), or trypsin/EDTA. (A). Representative Hematoxylin-Eosin-Saffranin (HES)-satined sections of lungs of mice are shown. Digitization of the whole slides was performed for each animal/treatment type. Histopathological analysis was done after delineation of metastases (dashed line) vs whole lung area (B) on each HES-stained section with the ImageJ software. The lung metastasis indices within each cohort of mice are expressed as fold decrease (+/−SEM) over untreated controls ***P<0.01. (C) Representative sections of CXCR4 immunostaining are shown.

To extend the biological relevance of these *in vitro* findings, an experimental model of lung metastasis was used [33]. In this model, mouse TC-1 cells, generated by transduction of C57BL/6 (B6) primary lung epithelial cells with a retroviral vector expressing HPV16 E6/E7 [33], are intravenously injected into C57BL/6 mice, resulting in a respiratory distress syndrome 15 days after injection. The morbidity of the animals was caused by dramatic, progressive metastatic lesions invading the lung parenchyma (‘Untreated Control’, Figure 3A and B). Such metastatic foci mainly comprised >90% CXCR4^+^ tumor cells (‘Untreated Control’, Figure 3C). The use of a monoclonal antibody blocking CXCR4 surface receptors prior to intravenous injection of TC-1 cells did not affect cell viability (98% living cells after incubation with the blocking antibody for 30 min.), but remarkably, almost completely abrogated tumor cell homing and invasion to the lung (‘Ab anti-CXCR4’; Figure 3A). In the antibody-pretreated group, less than 10 tiny tumor foci per section were observed 15 days after injection *versus* a dramatic tumor invasion in the control and non relevant antibody-treated groups (‘Ab anti-CD71’; Figure 3A). Quantification of these data was by calculation of the mean metastatic surface area measured in the cohort of animals used for each condition (Figure 3B, ‘Ab anti-CXCR4’, *** *P*<0.01). The remaining foci comprised CXCR4-positive tumor cells, suggesting that blockade was not total (Figure 3C, ‘Ab anti-CXCR4’). The specificity of these findings was then confirmed in three independent sets of control experiments. First, receptors were depleted from the surface of the cells by a 5 min trypsin/EDTA pre-incubation at 37°C. While this pre-treatment did not affect mass culture cell survival (data not shown), it almost fully abrogated tumor focus formation (n = 7/7 mice; ‘trypsin/EDTA panel’, Figure 3A, B, *** *P*<0.01). Second, pre-incubation of TC-1 cells with an irrelevant antibody (CD71) had no effect on the formation of TC-1 metastatic lesions, comparable to that in controls (n = 5/5 mice; Figure 3A and B; ‘Ab anti-CD71’). Third, histologic examination of the spleen showed normal morphology, indicating an absence of immune-related scavenging of the TC-1 cells/blocking antibody complex (n = 16/16 mice; Figure S2), suggesting that TC-1 cells were not eliminated by the immune system. Collectively, these experiments strongly suggested that pulmonary adhesion and invasion leading to subsequent proliferation of TC-1 foci into the lung is directly mediated by the CXCR4 pathway *in vivo*.

### Cidofovir decreases CXCR4 expression

**Figure 4:**
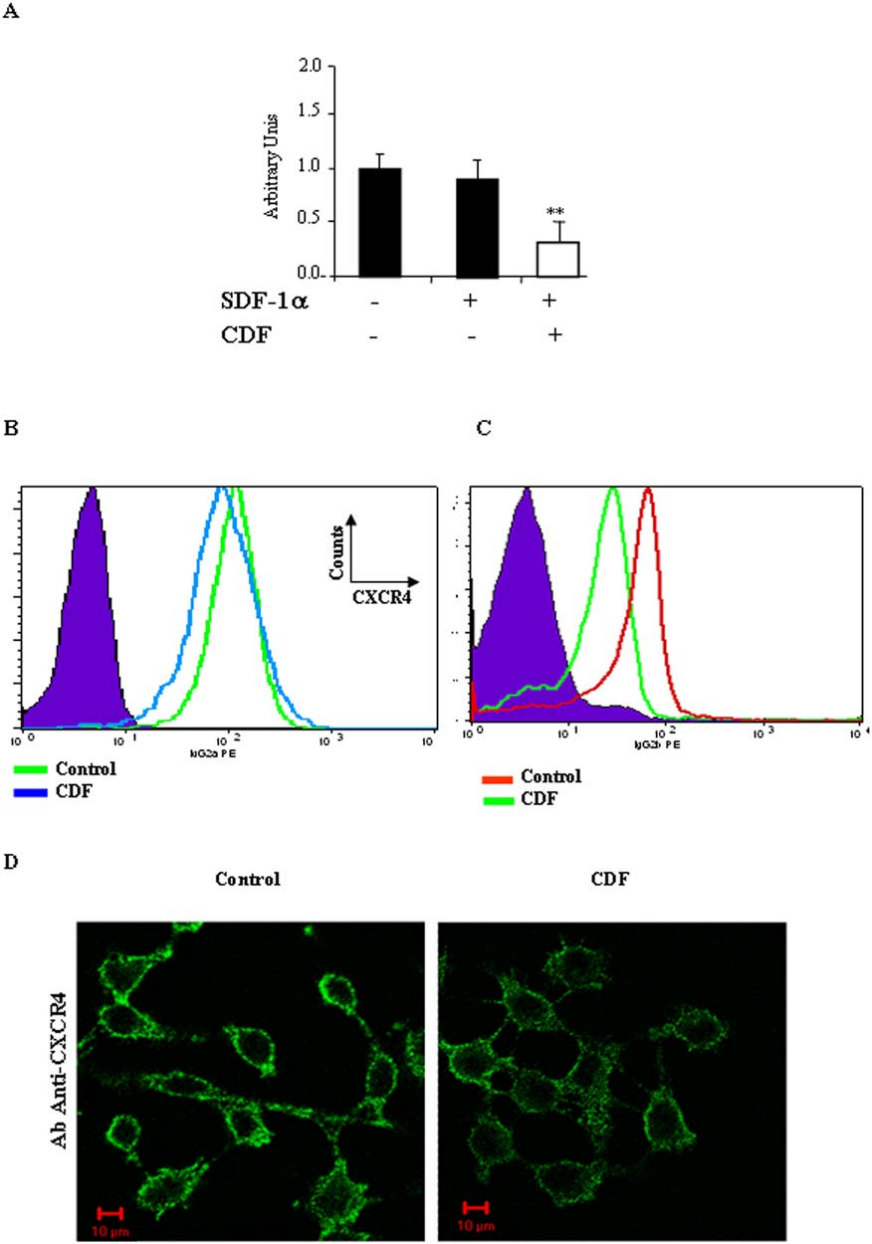
Cidofovir alters *CXCR4* gene and cell surface receptor expression. (A) Quantitative RT-PCR analysis of CXCR4 mRNA level in HeLa cells treated or not with SDF-1 and CXCR4- specific probe. **P<0.05. (B, C) FACS analysis of the cell surface expression of the receptor CXCR4 on HeLa (10^5^ cells/mL) (B) and (C) TC-1 cells (10^5^ cells/mL) treated or not with Cidofovir. Purple area = isotype control. (D) Subcellular localization of the CXCR4 receptor in HeLa studied by confocal microscopy using de MAB173 antibody from R&D System) at 1 µg/mL.

Because Cidofovir treatment inhibits cell invasion *in vitro*, we reasoned that Cidofovir might alter the abundance of CXCR4 mRNA and cell surface protein level. Q-RT-PCR analysis confirmed this assumption, showing a two-fold decrease in CXCR4 mRNA levels after TC-1 cellular incubation with Cidofovir (Figure 4A, ** *P*<0.05), whereas in the negative control, SDF-1α did not alter CXCR4 mRNA levels. FACS analysis confirmed that Cidofovir reduced CXCR4 receptors on the surface of both HeLa and TC-1 cells (Figure 4B: *P*<0.05 and Figure 4C: *P*<0.01). Immunofluorescence analysis confirmed the aforementioned data, showing both the expected exclusive membrane location of CXCR4 and its' reduced abundance on the cell surface after Cidofovir treatment (Figure 4D)

### Cidofovir inhibits experimental metastasis *in vivo*

We then examined whether pre-treatment of TC-1 cells with Cidofovir could also abrogate metastasis *in vivo*. Before to this, we excluded any non-specific anti-clonogenic effect of Cidofovir *in vitro* by clonogenic survival assay and *in vivo* by subcutaneous engraftment of TC-1 and subsequent monitoring of tumor growth (Figure S3). *In vitro*, the highest concentration of Cidofovir (10 µg/mL) reduced TC-1 clonogenic capacity by 22% (Figure S3A) and was used to pre-treat TC-1 cells before their subcutaneous injection. In mice injected with 4 million of untreated TC-1 cells, tumor engraftment defined by a 5 mm3 lesion took 10 days (Figure S3B) whereas delayed engraftment (23 days) was observed in mice injected with Cidofovir-treated TC-1 cells (Figure S3B). However the slopes of the tumor growth curves were similar in the untreated and Cidofovir-treated groups, suggesting that Cidofovir delayed TC-1 cells engraftment to the lung but has no effect on the clonogenic capacity of TC-1 *per se*.

**Figure 5:**
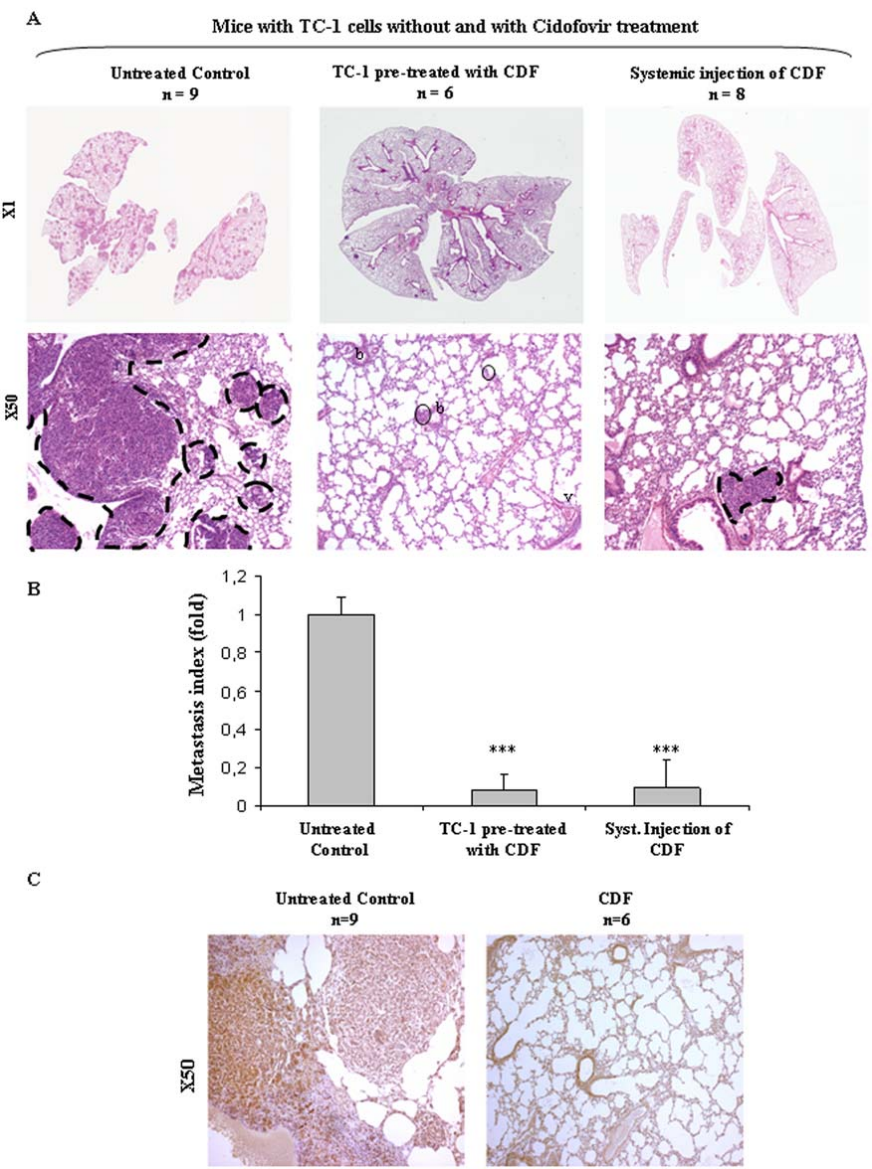
Cidofovir abrogates TC-1 cell lung metastasis *in vivo* in a CXCR4 dependent manner. In the two first groups, TC-1 cells were either untreated or pre-treated for 6 days with 10 µM Cidofovir prior to vein tail injection. In the third group, mice were systemically treated with Cidofovir (100 mg/Kg) 2 h before TC-1 cell injection and then on day at day 2, 4, 6, 8. Lungs were collected and processessed for histology. (A) Representative Hematoxylin-Eosin-Saffranin (HES)-stained sections of lungs of mice are shown. Metastases are delineated by a dashed line. b = bronchi, v = vessel (B) Quantitative assessment of lung macro-metastasis was performed as previously described ***P<0.01 as compared to untreated group. (C) CXCR4 immunostaining was performed as previously described.

We subsequently performed metastasis experiments. In the first set of experiments, mice were injected with TC-1 cells pretreated or not with Cidofovir (10 µg/mL). Notably, Cidofovir pre-treatment markedly abrogated both lung adhesion and invasion of TC-1 cells, completely in 4/6 mice, and in the two remaining mice, only two small microscopic metastatic foci were present 15 days after injection (both comprising 1–2 CXCR4+ tumor cells (Figure 5A, 5B middle panel, 5C, *** *P*<0.01). In a second set of experiments, Cidofovir (100 mg/kg) was systemically delivered to mice 2 h before IV injection of untreated TC-1 cells and mice were injected with Cidofovir for the 8 subsequent days. Interestingly, systemic Cidofovir treatment also decreased the number and size of TC-1 cell-induced metastatic foci: two mice out of eight exhibited lungs completely free of metastasis and in the remaining animals, metastatic foci were small with intra-vascular and intra-parenchymal locations. Quantification of such metastatic *versus* normal lung areas clearly showed the anti-metastatic efficacy of Cidofovir (Figure 5B, *** *P*<0.01).

### The Rho/ROCK pathway is activated by SDF1α and is inhibited by Cidofovir

**Figure 6:**
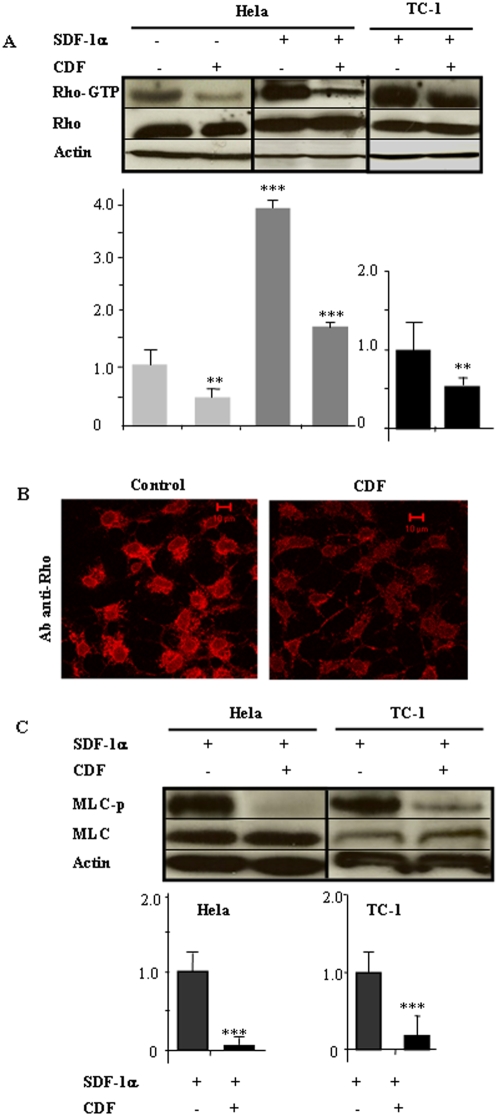
Cidofovir inhibits Rho/p160ROCK activation induced by SDF-1/CXCR4. (A) Active Rho was isolated by pull-down assay from HeLa and TC-1 cells after treatment with 100 ng/mL SDF-1 and 10 µM Cidofovir. Densitometric quantification was performed using ImageJ software. **P<0.05, ***P<0.01. (B) Membrane-associated Rho was studied by confocal microscopy. (C) p160ROCK activity was indirectly assessed by measuring myosin light chain phosphorylation (MLC-p). Densitometric analyses were performed using ImageJ software. ***P<0.01.

Because Rho/ROCK activation is involved in CXCR4 signaling, we examined its' possible activation by the SDF-1α ligand. In E6/E7 positive cell lines HeLa and TC-1, pre-incubation with SDF-1α resulted in clear activation of the Rho protein, as quantified by pull-down assay (Figure 6A, ** *P*<0.01). In contrast, cells exposed to Cidofovir demonstrated a significant decrease in both baseline and SDF-1α-activated levels of the Rho-GTP active form (Figure 6A, *** *P*<0.01). Although not quantitative, immunofluorescence assays confirmed pull-down result: SDF-1α-treated HeLa cells exhibited a membrane-associated Rho location, consistent with Rho's activation status; Cidofovir treatement decreased the fluorescence brightness (Figure 6B). We also indirectly examined ROCK activation by assaying the phosphorylation status of the myosin light chain (MLC), confirming that Cidofovir inhibited ROCK activity in both cell types (Figure 6C) *** *P*<0.01).

### ROCK inhibition partially blocks TC-1 metastasis

**Figure 7:**
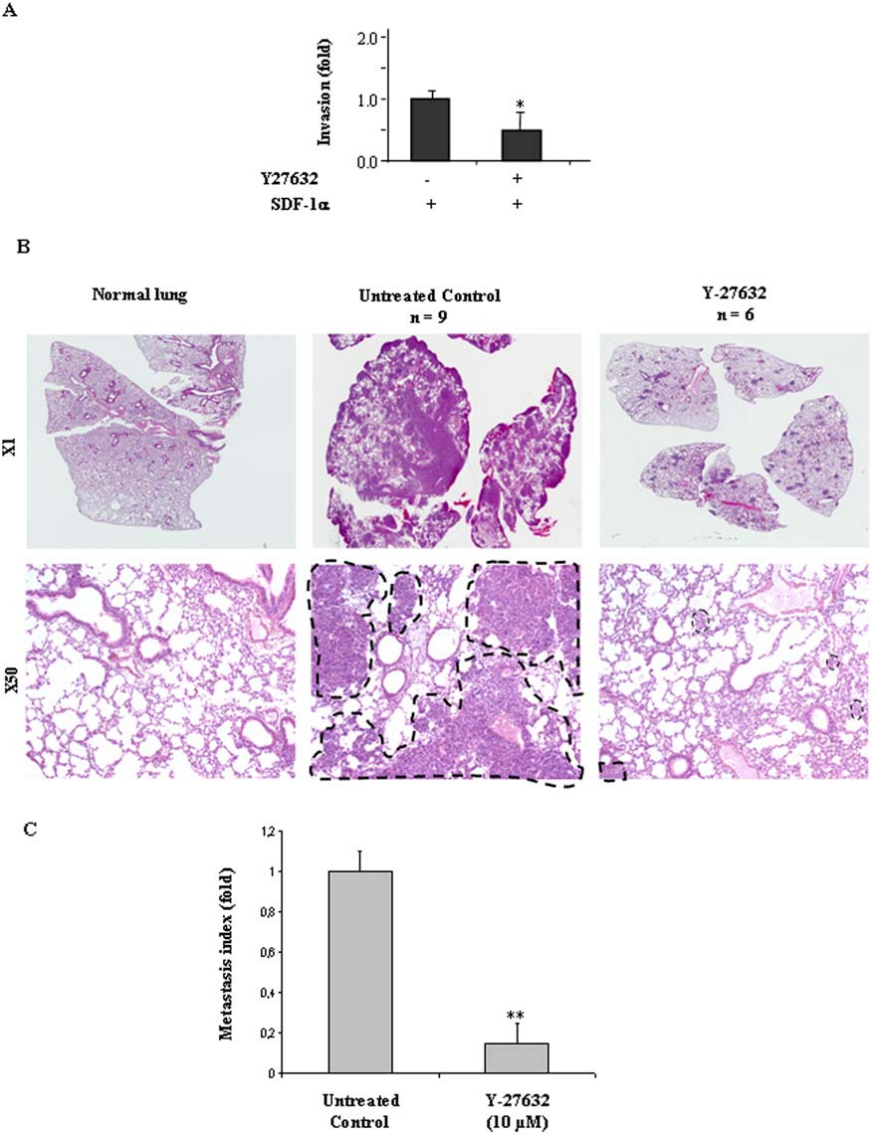
p160ROCK inhibition reduces HeLa migration *in vitro* and TC-1 cell lung metastasis *in vivo.* Matrigel assay were performed as previously described with HeLa cells pre-treated for 30 min 10 µM Y-27632. *P<0.05. (B) TC-1 cells were harvested and injected as previously described. Before injection to the mice, TC-1 cells were un-treated or pre-treated for 30 min. with 10 µM Y-27632. Lungs were collected, paraffin-embedded, fixed and cuted into sections. (A) Sections were stained with Hematoxylin-Eosin-Saffranin (HES) and (B) Quantitative assessment of lung macro-metastasis was performed as previously described. ***P<0.01 as compared to untreated group.

This last result suggested that the Rho/ROCK pathway may be involved in tumor cell invasiveness. We addressed this hypothesis *in vitro* and *in vivo* using a well established pharmacological inhibitor of ROCK, the small molecule Y-27632. Blockade of ROCK reduced both tumor cell invasion *in vitro* (Figure 7A) as well as pulmonary homing and colonisation *in vivo* (Figure 7B, C), supporting our former data. However, cellular Y-27632 exposure was less efficient at reducing matrigel invasion and metastasis than either CXCR4 blocking antibody or Cidofovir, suggesting that additional intracellular signaling is probably activated downstream of CXCR4 in E6/E7 expressing cells.

## Discussion

Clinical reports on patients with HPV positive tumors with distant metastasis in organs such as lung, liver and bone suggested an association between the presence of oncogenic human papillomaviral subtypes and the metastatic process [24], [25], [26]. More recent transfection/infection studies demonstrated that the oncoprotein E7 was a causative agent in early metastatic conversion [27], [34]. In the present study, we postulated that E6 and E7 expression could also strengthen tumor cell capability to home into distant metastatic sites in conjunction with microenvironmental stimuli, including the SDF-1α/CXCR4 axis. We developed *in vitro* and *in vivo* functional assays modeling the extravasation step of the metastatic process along with immunological, genetic and pharmacological approaches targeting CXCR4 or E6/E7 oncoproteins and showed a contribution of the SDF-1α/CXCR4/Rho/ROCK cascade and E6/E7 oncoproteins in experimental metastasis. In addition, our results suggested a new therapeutic strategy whereby HPV^+^ tumor cell homing and invasion may be prevented by an approved systemic agent, Cidofovir.

In the classical model of metastasis [35], a small subpopulation of permissive cells within a specific tumor microenvironment acquire genetic and epigenetic alterations that enable them to metastasize to distant sites after intravasation, transit in the blood or lymph, extravasation and growth. Extravasation typically occurs after attachment of circulating tumour cells to the vessel wall, directed by tissue-specific extracellular chemokines. The SDF-1α/CXCR4 axis belongs to this class of mediators and recent studies have shown that inhibition of CXCR4 with antibodies, peptide antagonists or siRNA all reduce metastasis in several solid tumors, including breast [8], [36], [37], [38], melanoma [12], [39], pancreas [40], prostate [41] and colon [42] cancers. Contribution of the SDF-1α/CXCR4 axis to the migration of cervix carcinoma cells was suspected; indeed a pro-migratory action of the SDF-1α has been reported in HeLa cells [43], [44]. In addition, transcriptional activation of the CXCR4 promoter is described in cervix carcinoma [45] and immunohistochemistry has shown high CXCR4 expression on cervix cancer cells [11]. Further implication of CXCR4 receptors in the metastatic process was extended *in vivo*. Inhibition studies with a CXCR-4-blocking-antibody almost completely abrogated pulmonary colonization *in vivo*. The current study showed that SDF-1α stimulated the invasive phenotype of human and rodent tumor cells. SDF-1α incubation stimulated the CXCR4 cascade without altering CXCR4 gene transcription, but activated one downstream signaling, *i.e.* the Rho/ROCK pathway. Findings are consistent with Bartolome *et al's* previous report, which showed that RhoA activity was enhanced in murine B16F10 melanoma cells upon SDF-1 stimulation [12] and involved the two Rho-GTPase guanine-nucleotide exchange factors Vav1 and Vav2 [13]. In addition, direct monitoring of ROCK activity (*i.e.* phosphorylation of the MLC) *in vitro*, and its pharmacological inhibition (*i.e.* Y-27632) *in vivo*, showed a contribution of ROCK activity to SDF-1 stimulation and lung homing of tumor cells expressing E6/E7. This last finding is not consistent with Charette *et al*'s report, which showed that inhibition of ROCK enhanced migration of normal keratinocytes transfected with E7. These discrepancies are probably due to the different methodologies used. Charette *et al.* used normal keratinocytes and an *in vitro* 2-D migration assay, which are more representative of initial steps of the metastatic process. In contrast, our data is mainly from *in vivo* experiments. Such an approach is more relevant to the pathophysiological process and relates to later steps of the metastatic process including organ homing and invasion. However, the fact that ROCK blockade was less efficient at metastasis inhibition than direct CXCR4 blockade, suggests involvement of other pathways downstream of CXCR4, such as the PI3 kinase/Akt pathway [44].

A significant question addressed in this work is the contribution of the E6 and E7 oncogenes in determining the extent of the invasive phenotype. Integration of papilliomaviruses into the host genome of cancer cells has been suggested to be one of the initial genetic events leading to metastasis [27], [34] and transfection of the oncoprotein E7 has been shown to trigger the early steps of the metastatic conversion in human keratinocytes *via* stimulation of the cyclin-dependant kinase inhibitor p27 and subsequent activation of the PI3K/Akt pathway [27]. In the present study, relationship between E6/E7 expression and the SDF-1α/CXCR4 axis was postulated. We showed that E6/E7 gene silencing or pharmacological inhibition using Cidofovir exhibited similar effects, disrupting the SDF-1-induced invasion process, decreasing CXCR4 expression and inhibiting both constitutive and SDF-1-induced Rho/ROCK activation. These results suggested that E6/E7 indeed contribute to the metastatic process and raise a rational for its abrogation in cervix cancer patients by using an anti-viral agent, Cidofovir, as a therapeutic tool. Potential non-specific toxicity of Cidofovir was carefully investigated *in vitro* and *in vivo* and at the concentration used, Cidofovir toxicity was found to be minimal, suggesting that its anti-metastatic action was not mediated through a simple killing process. While Cidofovir displays several intracellular actions such as cytostatic, radiosensitizing and anti-angiogenic actions, our group and others showed that Cidofovir and some of its derivates act preferentially on E6 and E7-expressing cells [29], [46], [47], [48], [49] inducing an S-phase block [50], [51] associated with decreased levels of E6 and E7, increased p53 and pRb levels. Consistently, the present study showed no anti-migratory effect of Cidofovir on HPV-negative B16F10 cells, but we report that the pro-invasive signals triggered by SDF-1α were occuring in HPV negative cells (*i.e.* B16F10), suggesting that SDF-1α/CXCR4 activation is not specific to E6/E7 expressing cells.

In summary, the present results show contribution of the SDF-1α/CXCR4/Rho/ROCK cascade in the metastatic process which is enhanced by the expression of E6 and E7 oncoprotein. These results show that E6/E7 act as enhancers of the metastatic process rather than initiators. Besides expression of E6 and E7 genes, additional events such as Ras activation could increase malignant conversion and tumor forming ability of HPV expressing cells [52], [53] and will worth careful investigation in the future. Finally, we showed for the first time an effective therapeutic inhibition of metastasis in E6/E7 expressing cells using Cidofovir in a dose-range suitable for clinical use. These data provided a compelling rational for investigation of a phase I clinical trial conducted at our Institution to assess Cidofovir toxicity in cervix patients (PHRC 2006).

## Materials and Methods

### Cell lines and treatments

HeLa human cervix carcinoma (HPV18^+^) were cultured in DMEM supplemented with 10% FCS, 50 U/ml penicillin/streptomycin, 2 mM L-glutamine, 1 mM sodium pyruvate and 2 mM non-essential amino acids (CCL-2, Promochem, France). Mouse lung carcinoma TC-1 cells (HPV16^+^) were cultured in RPMI 1640 supplemented with 10% FCS, 50 U/ml penicillin/streptomycin, 2 mM L-glutamine addition of 1 mM sodium pyruvate, 2 mM non-essential amino acids and 0.4 mg/ml G418 (CRL-2785, Promochem, France). TC-1 cells were initially obtained by co-transformation of primary C57BL/6 mouse lung epithelial cells with HPV-16 E6 and E7 and activation of Ras oncogene [33] and used in the present study to generate lung metastasis in mice as previously described [33]. The TC-1 metastasis model was used because HeLa cells do not form spontaneous metastases when injected into nude mice. Mouse melanoma B16F10 cells are HPV-negative and were used to monitor the specificity of Cidofovir action. They were cultured in DMEM supplemented with 10% FCS, 50 U/ml penicillin/streptomycin, 2 mM L-glutamine.

Cells were collected by gentle scraping to maintain the CXCR4 receptor at the cell surface. CXCR4 blocking antibodies were, respectively, 12G5 for HeLa cells (10 µg/mL; R&D system) and 2B11 for TC-1 and B16F10 cells (10 µg/mL; R&D system). Positive and negative control experiments were performed by pre-incubating scraped cells with an irrelevant antibody (monoclonal, CD71, 10 µg/mL, BD Pharmingen) or trypsin/EDTA (Gibco). To shut-down E6/E7 expression, HeLa, TC-1 cells and B16F10 were treated *in vitro* with 10 µM Cidofovir (Pharmacia Upjohn, Guyancourt les Yvelines, France) for 6 days. Concentration and timing have been optimized in previous experiments [29], [47] and efficiently switched-off E6/E7 expression. Similarly, scraped cells were treated with Y-27632 10 µM (Tocris) for 30 min before seeding or IV injection. In addition, E6/E7 expression was stably knocked-down in HeLa cells using the pEBVsiRNA as previously described [30]. Silenced HeLa cells were termed as HeLa KD (*knock down*) cells. For each gene, 3 vectors were constructe, selected with hygromycin and sub-cultured in presence of hygromycin (125 µg/mL, Invitrogen). Each siRNA sequence and its position are indicated Table 1. Inhibition of E6 and E7 was checked by Q-RT-PCR as previously described [47], E6 primers were as follows: Fwd *5′- gcg acc cta caa gct acc tg -3′* and Rev *5′- aga cag tat acc cca tgc tg -3′* and E7 primers were as follows: Fwd *5′- aga aac cca gct gta atc -3′* and Rev *5′- tta tgg ttt ct gaga aca ga -3′* (Perkin-Elmer Corp./PE Applied Biosystems).

**Table 1:** pEBVsiRNA sequences.

pEBVsiRNA plasmid	Position (nt)
	Sequence
pEBVsiHPV18E6-104R	104–122
	5′-GCAAGACAGTATTGGAACT-3′
pEBVsiHPV18E7-609R	609–627
	5′-CAAGACATTGTATTGCATT-3′
pEBVsiHPV18E7-698R	698–716
	5′-CGATGAAATAGATGGAGTT-3′
pEBVcontrol	[30]

### Western-blot analysis

Western-blot analysis was conducted as already described [47]. Briefly total protein was isolated, subjected to SDS polyacrylamide gel electrophoresis and electrotransfered onto nitrocellulose membranes. Monoclonal antibodies against E6 (1∶2500, clone C1P5, Abcam) was used as well as anti-β-actin antibody (clone AC-15, Sigma) to control protein loading.

### Cytotoxicity

Cell survival after Cidofovir treatment was monitored using trypan blue exclusion and MTT assays. Apoptosis was monitored by TUNEL assay (Roche) according to manufacturer instructions and subsequent confocal observation. Clonogenic survival assay were also performed *in vitro* using increasing concentration of Cidofovir (1–10 µM) and *in vivo* monitoring tumor growth. Cidofovir anti-clonogenic action was monitored by sub-cutaneous injection of TC-1 and follow-up of tumor engraftment and growth. Mice were divided into 3 groups (n = 6mice/group): 4 million of untreated TC-1, 4 million of Cidofovir-pretreated TC-1 and 8 million of Cidofovir-pretreated TC-1.Toxicity of Cidofovir was assessed to make comparison between control and Cidofovir-treated cells possible. The number of cells used in the various experiments was corrected according to these results to be sure that the exact same number of living cells was seed in control and Cidofovir-treated cells.

### Tumor cell invasion assay

As an *in vitro* model system for metastasis, we performed matrigel invasion assay using transwell chambers (24-well format, 8-µm pore size; thin matrigel layer; BD Falcon). Human recombinant CXCL12/SDF-1 (100 ng/mL; R&D Systems) in FCS free medium was added to the bottom chamber to induce cells' invasion through the matrigel. Cells were pre-treated using: 1) CXCR4-blocking antibody (10 µg/mL) and 2) Cidofovir and 3) Y-27632. The antibody concentration was based on Li et *al's* previous report [8]. For each treatment group 5×10^5^ cells were seeded into the top chamber. Matrigel invasion chambers were incubated for 24 hours at 37°C and non-invading cells removed from the top of the matrigel using a cotton-wool swab. Invading cells at the bottom of the chamber were fixed and stained with crystal violet. The invasion rate was determined by counting the crystal violet-stained cells. Results were expressed as the mean number of invaded cells−/+SD. Similar procedure was used for E6^KD^ and E7^KD^ HeLa cell lines. Three independent experiments were performed, with three chambers in each treatment group.

### Metastasis experiment *in vivo*, histology and immunohistochemistry

Experiments were performed with 10-week-old female C57BL/6 mice (Harlan, France). Experiments were conducted under the French regulations for animal experimentation (Ministry of Agriculture ActNo.87-848, 19^th^ of October, 1987) and received ethics approval.TC-1 cells were harvested by gentle scraping in FCS free RPMI 1640 to obtain single-cell suspension. Cells were pre-incubated 30 min with CXCR4-blocking antibody, irrelevant antibody (CD71) and trypsin/EDTA respectively. To shut-down E6/E7 expression, TC-1 cells were pre-incubated for 6 days with Cidofovir as previously described. One million TC-1 cells in 0.1 ml FCS-free medium were injected into mouse tail veins using a 25-gauge needle. Mice were divided into 6 groups according to the pre-treatment cells received (n = 5–9 mice/group): carrier only, CXCR4-blocking antibody, irrelevant antibody (CD71), trypsin/EDTA, Cidofovir and Y-27632. The anti-metastatic efficacy of Cidofovir was also assessed after systemic administration of Cidofovir at 100 mg/kg, a non-toxic dose used in previously experiments [29]. 8 mice received intra-peritonal injection of Cidofovir 2 h prior IV injection of 1 million TC-1 cells and subsequent injection at day 2, 4, 6, 8. At the end of 15 days follow up and whatever treatments they had all mice were killed [33]. Lungs and spleen were collected for histology and immunohistochemistry. Spleen was collected to monitor possible immune-related activation. Organs were fixed in Finefix (Milestone medical, Italy), paraffin embedded and cut into 4 µm sections. Sections were stained with Hematoxilin-Eosin-Saffranin (HES) and examined using conventional light microscopy. Assessment of lung macro-metastasis was performed on HES-stained section digitized with a slide scanner (Super-coolscan 8000 ED; Nikon) and analyzed with the ImageJ software downloaded from http://rsb.info.nih.gov/ij/ to determine the total metastatic surface per lung of each mouse. CXCR4 immunostaining was performed using a goat polyclonal antibody raised against CXCR4 and incubated overnight at 4°C (n°201-819; 1∶300; Alexis Biochemicals). Primary antibody incubation was followed by incubation with rabbit anti-goat serum for 30 min. (1∶50, Dako), streptavidin-HRP for 30 min. (1∶100, Dako) and revealed with DAB substrate (Dako).

### FACs analysis

FACs analysis of CXCR4 surface expression was studied using a specific CXCR4 receptor monoclonal antibody 12G5-PE for HeLa cells (10 µg/mL; BD Pharmingen, Le Pont-De-Claix – France) and 2B11-PE for TC-1 (10 µg/mL; R&D system). Cells were stained with appropriate dilutions of the antibodies or isotype-matched controls. After one wash, cells were suspended in phosphate-buffered saline (PBS), kept at 4°C, and analyzed on a FACsort (Becton, Dickinson) with the CellQuest Pro-software package.

### Confocal microscopy analysis

Immunofluorescence was evaluated by laser scanning confocal microscopy (Zeiss LSM510, Carl Zeiss SAS, France) after fixation of sub-confluent HeLa cells with paraformaldehyde 1% and incubation with appropriate primary and secondary antibodies. Labeling was performed with a primary mouse anti-CXCR4 monoclonal antibody (MAB173; 1∶250, R&D Systems, Abingdon – UK) and a secondary goat F(ab′)2 anti-mouse IgG2b FITC -coupled antibody (1∶500; Santa Cruz, Tebu bio, France), anti-Rho A/B/C (H-70) rabbit IgG polyclonal antibody (1∶300; Santa Cruz, Tebu-bio, France) with secondary antibody Alexa Fluor-546 conjugated goat anti-rabbit IgG (1∶300) (Molecular Probes, Eugene, OR).

### Real-time PCR

Total RNA was isolated from cells using an RNeasy mini kit according to the manufacturer's instructions (QIAGEN, Hilden, Germany). The quality of RNA was assessed using a bioanalyser (Agilent, Les Ulis, France) and 1 microgram of total RNA was reverse transcribed as described [47]. CXCR4 primers were as follows: TaqMan *5′- cag cag gta gca aag tga cgc cga -3′*, Fwd *5′- gtt tgt tgg ctg cgg -3′* and Rev *5′- gtc ata gtc ccc tga gcc ca -3′* (Perkin-Elmer Corp./PE Applied Biosystems). Real-time quantitative PCR was achieved according to the manufacturer's instructions and normalized to 18S ribosomal RNA (Perkin-Elmer Corp./PE Applied Biosystems).

### Rho and ROCK activity assay

For the Rho pull down assay, cells were incubated with 100 ng/mL of recombinant human SDF-1α ligand (R&D Systems) 15 min before protein collection. The Rho-GTP form was isolated by pull-down assay (Pierce, Perbio, France) according to the manufacturer's instructions. Anti-Rho A/B/C (H-70) rabbit IgG polyclonal antibody from Santa-Cruz (1∶1000) was used to detect total Rho protein, whereas anti-Rho mouse monoclonal antibody from Pierce was used for Rho-GTP detection (1∶500, Pierce, Perbio – France).

ROCK activity was indirectly assessed by monitoring the phosphorylation status of the MLC (Myosin light chain). Cells were gently scraped from flasks in cold PBS, with a protease cocktail inhibitor (Roche Diagnostics, GmbH, Germany) and anti-phosphatase (orthovanadate 5 mM), then lysis buffer (10 mM HEPES pH = 8, 0.1 mM EGTA pH = 8, 0.01 M KCl, 1 mM DTT, 0.62% NP40, protease inhibitor and orthovanadate 5 mM) was added. Protein concentrations were assessed by Bradford assay (Bio-Rad, Marnes-la-Coquette, France) and Western blots were performed as described [47]. Anti-MLC II rabbit polyclonal was from Santa Cruz (1∶500; Tebu-bio, France) and anti-MLC-phospho (Ser19) rabbit polyclonal from Stressgen (1∶400; France). Anti-GAPDH mouse polyclonal (1∶5000, Biodesign, France) was used as loading control.

### Statistical analyses

All values are reported as mean (SEM). Data were analysed using one way ANOVA and the Student-Newman-Keuls test.

## Supporting Information

Figure S1 (0.43 MB TIF)

Figure S2 (5.97 MB TIF)

Figure S3 (0.77 MB TIF)
