# Supplementary figures and images for: Novel Anti-Metastatic Action of Cidofovir Mediated by Inhibition of E6/E7, CXCR4 and Rho/ROCK Signaling in HPV+ Tumor Cells
Source: PLoS One. 2009 Mar 26;4(3):e5018. doi: 10.1371/journal.pone.0005018 (PMC2657827; doi:10.1371/journal.pone.0005018)

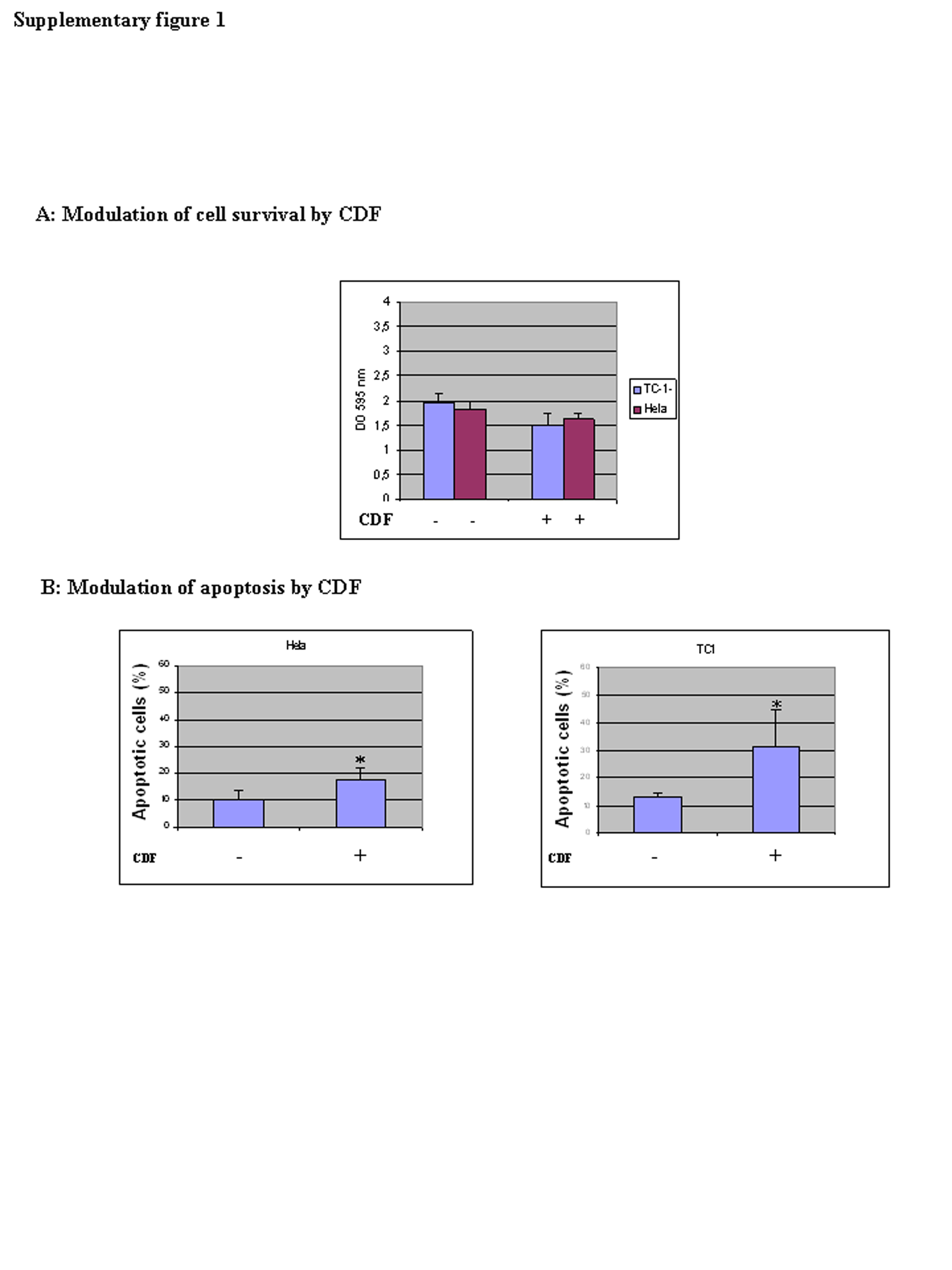

Supplement: Figure S1 — (0.43 MB TIF) [file pone.0005018.s001.tif]

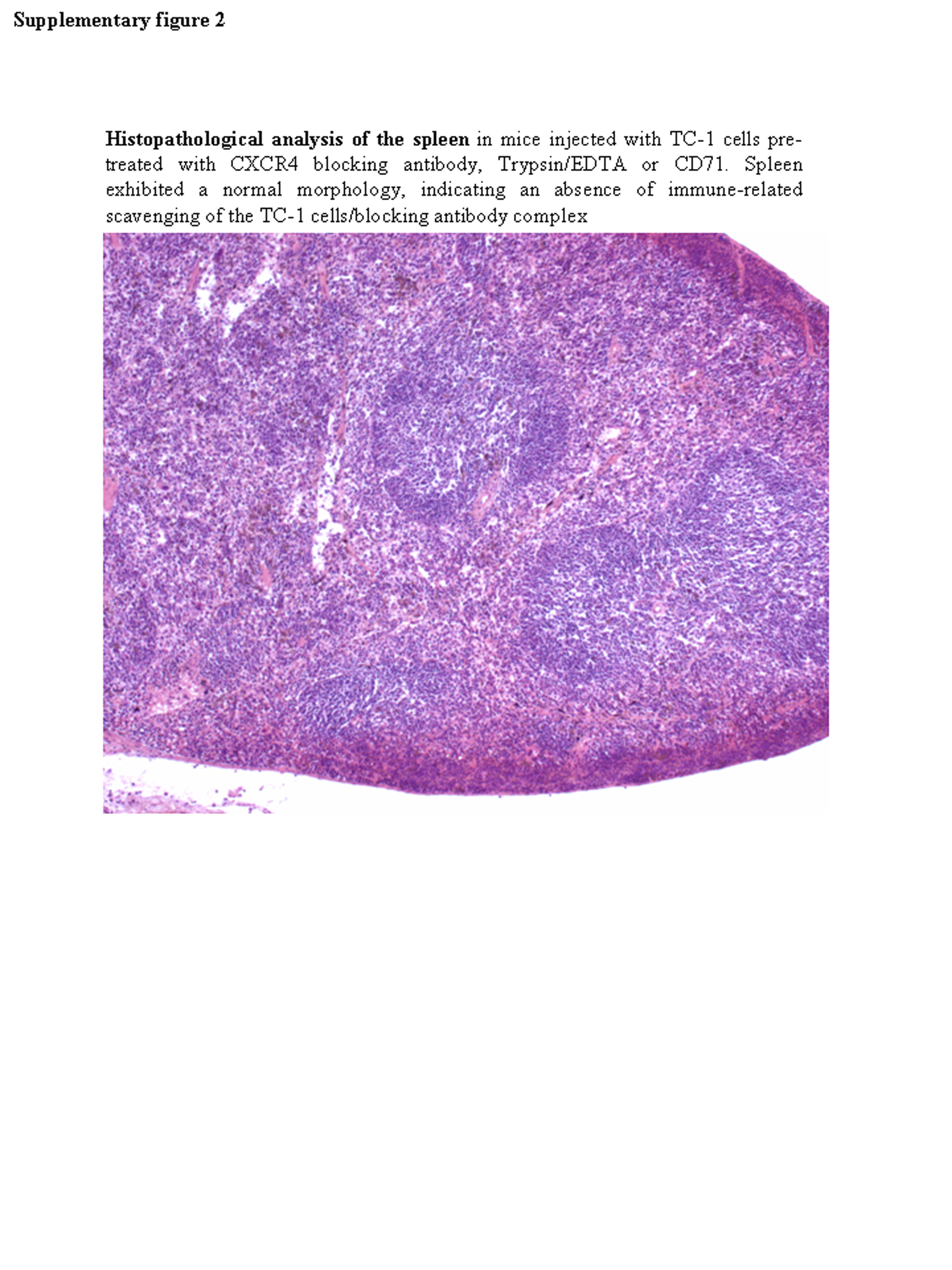

Supplement: Figure S2 — (5.97 MB TIF) [file pone.0005018.s002.tif]

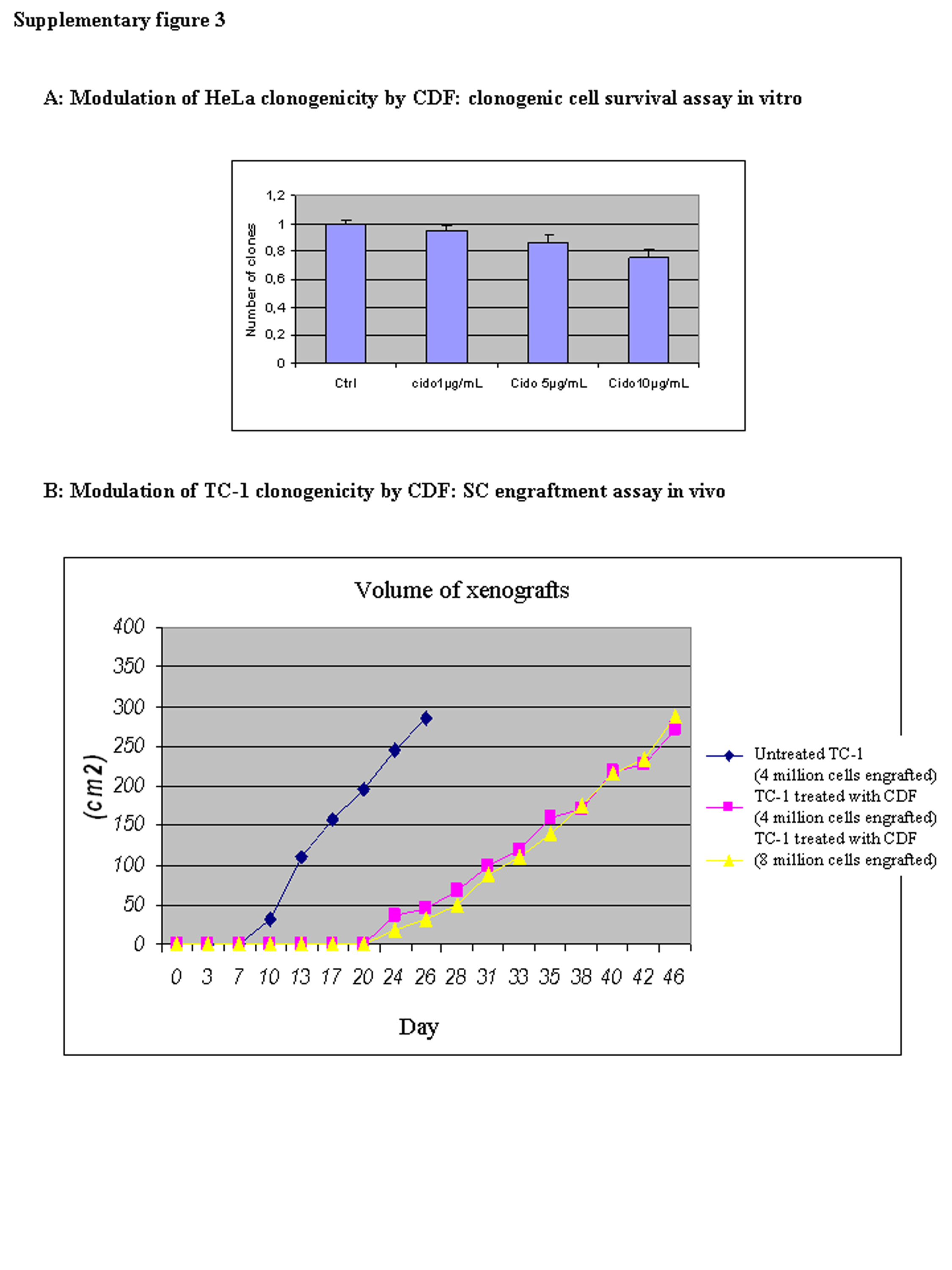

Supplement: Figure S3 — (0.77 MB TIF) [file pone.0005018.s003.tif]
